# Using a vector pool containing variable-strength promoters to optimize protein production in *Yarrowia lipolytica*

**DOI:** 10.1186/s12934-017-0647-3

**Published:** 2017-02-17

**Authors:** Rémi Dulermo, François Brunel, Thierry Dulermo, Rodrigo Ledesma-Amaro, Jérémy Vion, Marion Trassaert, Stéphane Thomas, Jean-Marc Nicaud, Christophe Leplat

**Affiliations:** Micalis Institute, INRA-AgroParisTech, UMR1319, Team BIMLip: Integrative Metabolism of Microbial Lipids, Université Paris-Saclay, domaine de Vilvert, 78350 Jouy-en-Josas, France

**Keywords:** *Yarrowia lipolytica*, Protein production, RedStar2, Glucoamylase, Xylanase, Hybrid promoters

## Abstract

**Background:**

The yeast *Yarrowia lipolytica* is an increasingly common biofactory. To enhance protein expression, several promoters have been developed, including the constitutive *TEF* promoter, the inducible *POX2* promotor, and the hybrid hp4d promoter. Recently, new hp4d-inspired promoters have been created that couple various numbers of UAS1 tandem elements with the minimal *LEU2* promoter or the *TEF* promoter. Three different protein-secretion signaling sequences can be used: preLip2, preXpr2, and preSuc2.

**Results:**

To our knowledge, our study is the first to use a set of vectors with promoters of variable strength to produce proteins of industrial interest. We used the more conventional *TEF* and hp4d promoters along with five new hybrid promoters: 2UAS1-p*TEF*, 3UAS1-p*TEF*, 4UAS1-p*TEF*, 8UAS1-p*TEF*, and hp8d. We compared the production of RedStar2, glucoamylase, and xylanase C when strains were grown on three media. As expected, levels of RedStar2 and glucoamylase were greatest in the strain with the 8UAS1-p*TEF* promoter, which was stronger. However, surprisingly, the 2UAS1-p*TEF* promoter was associated with the greatest xylanase C production and activity. This finding underscored that stronger promoters are not always better when it comes to protein production. We therefore developed a method for easily identifying the best promoter for a given protein of interest. In this gateway method, genes for YFP and α-amylase were transferred into a pool of vectors containing different promoters and gene expression was then analyzed. We observed that, in most cases, protein production and activity were correlated with promoter strength, although this pattern was protein dependent.

**Conclusions:**

Protein expression depends on more than just promoter strength. Indeed, promoter suitability appears to be protein dependent; in some cases, optimal expression and activity was obtained using a weaker promoter. We showed that using a vector pool containing promoters of variable strength can be a powerful tool for rapidly identifying the best producer for a given protein of interest.

**Electronic supplementary material:**

The online version of this article (doi:10.1186/s12934-017-0647-3) contains supplementary material, which is available to authorized users.

## Background

Increasing the efficiency of heterologous gene expression is a major goal for the agrifood, bioconversion, and pharmaceutical industries as they have a growing need for recombinant proteins. Expression systems using yeasts present several advantages: yeasts are easy to manipulate, they are unicellular organisms with rapid growth rates, and they are eukaryotes that can incorporate post-translational modifications. In addition to the more conventional *Saccharomyces cerevisiae* [1], alternative model species are also used as biofactories, including *Pichia pastoris, Hansanula polymorpha*, *Kluyveromyces lactis*, *Kluyveromyces marxianus* [2–5], and *Yarrowia lipolytica* [6, 7].

Production systems exploiting *Y. lipolytica* have several advantages [7, 8]. First, *Y. lipolytica* is a non-pathogenic organism that can grow on a diversity of substrates. Second, the products of several *Y. lipolytica*-based processes have received the “generally recognized as safe” (GRAS) designation from the FDA. Third, *Y. lipolytica* has a naturally strong secretory ability [7, 8] and demonstrates weak protein glycosylation [9].

Several genetic tools are available to enhance protein expression in *Y. lipolytica*. Indeed, integrative expression cassettes containing different markers, such as *LEU2*, *URA3, ADE2,* and *LYS5*, have been constructed. They can be used to transform competent auxotrophic strains of *Y. lipolytica*. Moreover, several promoters are also available, including the constitutive *TEF* promoter, the constitutive and hybrid hp4d promoter, and the inducible *POX2* and *LIP2* promoters [10–14]. In addition, several transformation methods have been developed to optimize the transformation rate [15–17]. Currently, the lithium-acetate method is the most common, whether the goal is to inactivate endogenous genes or to transform expression cassettes [18]. All of these tools have been successfully used in *Y. lipolytica* to produce such proteins as xylanase, lipase, leucine aminopeptidase II, human interferon, α2b endoglucanase II, and cellobiohydrolase II [6, 9, 14, 19, 20]. Past studies have also identified at least three sequences that can be used to optimize protein secretion in *Y. lipolytica*: preLip2, preXpr2, and preSuc2 [6, 14, 21–24].

Several studies have suggested that *Y. lipolytica* is better than *P. pastoris* at producing heterologous proteins [20, 25]. Indeed, Nars and colleagues [25] found that, as opposed to *P. pastoris*, *E. coli*, or simple free cells, *Y. lipolytica* was the best candidate for generating extracellular Lip2 because it can form a stable isotope-labeled version of the protein. Boonvitthya and colleagues [19] compared endoglucanase II and cellobiohydrolase II production in *Y. lipolytica* and *P. pastoris*. In YT medium, *Y. lipolytica* produced up to 15 mg/L of endoglucanase and 50 mg/L of cellobiohydrolase. Furthermore, the enzymes produced by *Y. lipolytica* had higher levels of specific activity than did their counterparts in *P. pastoris*. Finally, it has been found that *Y. lipolytica* has weaker protein glycosylation than does *P. pastoris* [9].

One of the first strong constitutive promoters was developed by Novo, using the *TEF1* gene, which encodes the translation elongation factor-1α [10]. Later, Madzak and colleagues [26] identified the upstream activating sequence UAS1 in the *XPR2* gene (which encodes the secreted alkaline extracellular protease). This discovery led to the development of the hp4d promoter, which is based on the minimal *LEU2* promoter and contains four UAS1 tandem elements; with this promoter, expression increases as the number of UAS1 tandem elements increases. More recently, several research groups have used this basic model (i.e., multiple UAS tandem elements associated with a core promoter) to develop improved promoters [27–29]. It has been found that the core promoter and the upstream activating sequence (i.e., the UAS1 tandem elements) act independently and that, as previously noted, promoter strength increases with the number of UAS1 tandem elements. Shabbir Hussain and colleagues [29] showed that promoter strength can be fine-tuned by engineering the sequences of the TATA box, the core promoter, or the upstream activating region. To quantify promoter strength, they used fluorescent proteins and β-galactosidase assays.

However, to our knowledge, no study to date has used these UAS1-based promoters to produce proteins of industrial interest. Here, we used two conventional promoters, p*TEF* and hp4d, as well as five new hybrid promoters of our own construction. To create the latter, we added two, three, four, or eight UAS1 tandem elements to p*TEF*; we also added four tandem elements to hp4d. Promoter strength in transformed *Y. lipolytica* strains was quantified using RedStar2, a fluorescent protein, as a reporter; we also analyzed the production of secreted *Aspergillus niger* glucoamylase (GA) and xylanase C (XlnC). GA is a glucan 1,4-alpha-glucosidase that belongs to the glycosyl hydrolase family. It catalyzes the degradation of starch and other complex sugars, releasing d-glucose. GA is largely used to produce biolipids and bioethanol from starch or lignocellulosic materials [30, 31]. XlnC is a beta-1,4-beta-xylanase that breaks down hemicellulose, a component of plant cell walls, releasing xylose. The paper, textile, and pet-food industries are major consumers of xylanase.

Our results revealed that optimal protein expression, secretion, and activity are not always correlated with promoter strength. Consequently, we developed a simple method for improving protein expression that involves the use of a pool of vectors containing promoters of variable strength.

## Methods

### Yeast strains, growth media, and culture conditions

The *Y. lipolytica* wild-type strain W29 (ATCC20460) was used as the basis for all the *Y. lipolytica* strains built in this study (see Additional file 1: Table S1 for the full list). The auxotrophic strain Po1d (Leu^−^ Ura^−^) has previously been described by Barth and Gaillardin [19]. *Escherichia coli* strain DH5α was used to construct the plasmids, except in the case of vectors containing *ccdB*, for which *E. coli* strain DB3.1 was used. *E. coli* growth media and culture conditions have been previously described by Sambrook and colleagues [32], and those for *Y. lipolytica* have been described by Barth and Gaillardin [15]. Rich medium (YPD) and minimal glucose medium (YNB) were prepared as described elsewhere [33]. The YPD medium contained 10 g/L of yeast extract (Difco, Paris, France), 10 g/L of Bacto Peptone (Difco, Paris, France), and 10 g/L of glucose (Sigma Aldrich, Saint-Quentin Fallavier, France). The YNB medium contained 1.7 g/L of yeast nitrogen base without amino acids and ammonium sulfate (YNBww; Difco, Paris, France), 10 g/L of glucose (Sigma), 5.3 g/L of NH_4_Cl, and 50 mM phosphate buffer (pH 6.8). This minimal medium was supplemented with uracil (0.1 g/L) and/or leucine (0.1 g/L) as necessary. YP_2_D_4_ medium contained 10 g/L of yeast extract (Difco, Paris, France), 20 g/L of Bacto Peptone (Difco, Paris, France), and 40 g/L of glucose (Sigma Aldrich, Saint-Quentin Fallavier, France). Solid media were created by adding 1.6% agar.

### Plasmid and strain construction

**Fig. 1:**
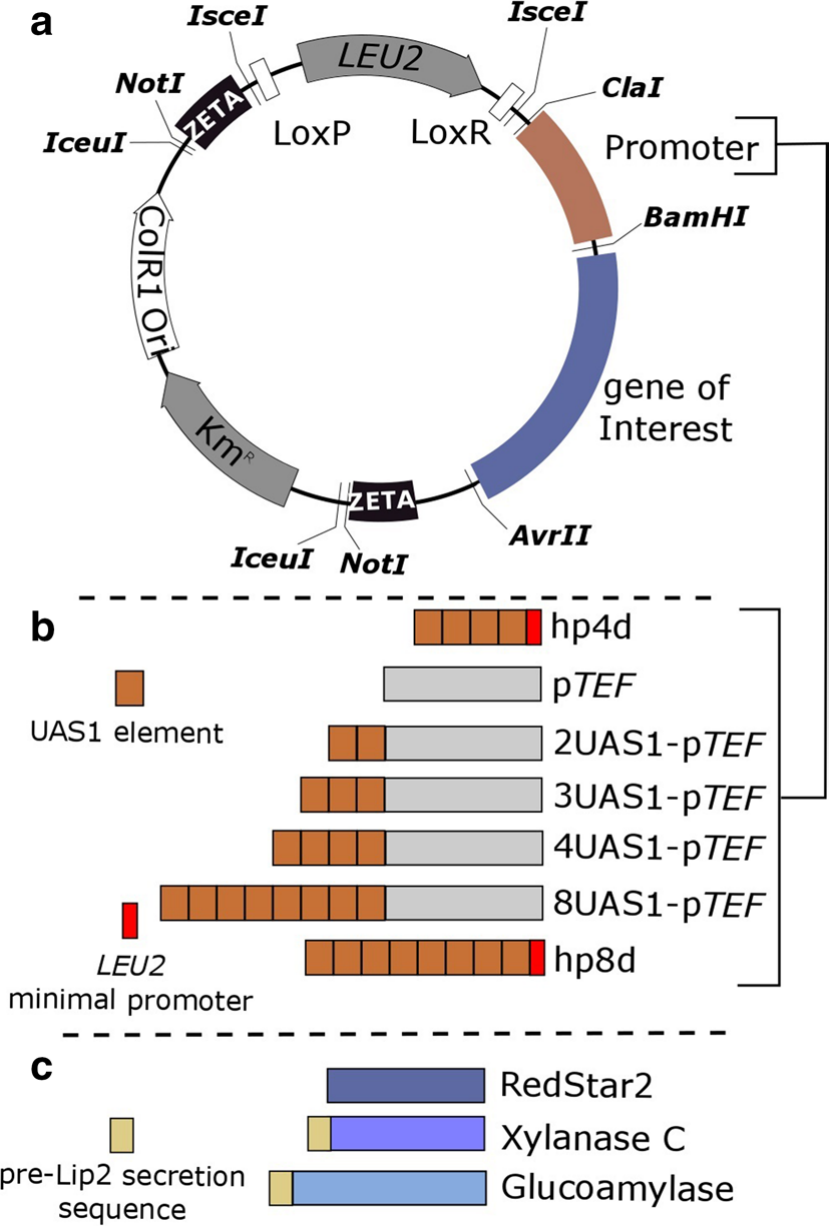
Schematic representation of plasmid construction. **a** Representation of the expression vector. The zeta region allows random insertion in the *Y. lipolytica* genome. LoxP and LoxR were used to rescue the marker. **b** Representation of the hybrid promoters containing p*TEF* (in *gray*), the UAS1 tandem elements (in *brown*), and the TATA box of the *LEU2* promoter (in *red*). **c** Representation of the genes of interest (in various *shades of blu*e); the preLip2 secretion sequence was present (in *yellow*) or absent

The structure of the plasmids constructed in this study was typical of that of the expression vector JMP62 [6] (Fig. 1a). The plasmids contained an excisable marker (the *I*-*sce*I fragment flanked by LoxP/LoxR [37]), and the promoter and gene of interest were carried in the *Cla*I-*Bam*HI and *Bam*HI-*Avr*II fragments, respectively. The zeta region for expression cassette integration was flanked by the *Not*I site, which is involved in the release of the expression cassette prior to transformation. Plasmid and strain construction are described in Additional file 2: Figure S1. In most cases, the genes of interest were introduced by digesting the corresponding donor plasmid using *Bam*HI-*Avr*II (Additional file 2: Figure S1a). Promoter exchange was performed by digesting the donor plasmid using *Cla*I-*Bam*HI; *Cla*I was used to insert the modified promoter (Additional file 2: Figure S1b).

The two, three, or four UAS1 tandem element fragments were amplified by PCR using HYB-*ClaI*3′Hp4d5′ and HYB-*BstbI*5′Hp4d3′ as primers (Table 1; Additional file 2: Figure S1). The corresponding fragments were ligated into pCR4Blunt-TOPO^®^ in accordance with the manufacturer’s instructions (Invitrogen, Saint-Aubin, France).

**Table 1:** List of primers used in this study

Primer	Sequence	Use
HYB-ClaI3′Hp4d5′	CCCTACATCGATACGCGTGC	Hybrid promoter construction
HYB-BstbI5′Hp4d3′	CCTTCGAACGCACTTTTGCCCGTGATCAG	
GATO_Amont_ClaI_for	CCCTGTTATCCCTAGAATCGAT	Verification of plasmid construction and insertion into the *Y. lipolytica* genome
GATO_Aval_AvrII_rev	TTAGATACCACAGACACCCTAG	
GATO_pTEF_BamHI_for	AACTCACACCCGAAGGATCC	
GATO_HP4d_BamHI_for	GAACCCGAAACTAAGGATCC	
YFP-pool-Fw	CACCATGGTGAGCAAGGGCGAGGAGC	Insertion of YFP gene into pENTR™/D-TOPO^®^
YFP-pool-Rv	TTACTTGTACAGCTCGTCCATGCC	
Amy-pool-Fw	CACCATGAAGCTGTCTACCATTCTG	Insertion of α-amylase gene into pENTR™/D-TOPO^®^
Amy-pool-Rv	TCAAATCTTCTCCCAAATAGCG	
1529BamHIcorrigéF	CCTTGTCAACTCACACCCGAAGGATCCATCACAAGTTTGTAC	Addition of a *Bam*HI site close to the promoter in JMP1529 to obtain JMP3030
1529BglIIcorrigéR	TCTGGCTTTTAGTAAGCCAGATCTACGCGTTTACGCCCCGCC	
1529BamHIcorrigéR	GTACAAACTTGTGATGGATCCTTCGGGTGTGAGTTGACAAGG	
qPCR_XlnCF	CGAGCTGCCGATCCCAATGCC	qPCR related to the XlnC gene
qPCR_XlnCR	GCTCCACCGCCTGCAGACA	
qPCR_YALI0D08272F	AGGCCCAGTCCAAGCGAGGT	qPCR related to the actin gene
qPCR_YALI0D08272R	TCGGTGAGCAGGACGGGGTG	

*GA* was cloned into the JMP2482, JMP2484, JMP2397, JMP2607, JMP2471, and JMP2473 plasmids at the *Bam*HI and *Avr*II restriction sites, yielding JMP3781 (*LEU2*ex 2UAS1-p*TEF*-*GA*), JMP3782 (*LEU2*ex 3UAS1-p*TEF*-*GA*), JMP3783 (*LEU2*ex 4UAS1-p*TEF*-*GA*), JMP3784 (*LEU2*ex 8UAS1-p*TEF*-*GA*), JMP3785 (*LEU2*ex hp4d-*GA*), and JMP3786 (*LEU2*ex hp8d-*GA*), respectively.

*XlnC* was cloned into the JMP2482, JMP2484, JMP2397, JMP2607, JMP2471, and JMP2473 plasmids at the *Bam*HI and *Avr*II restriction sites, yielding the JMP3096 (*LEU2*ex 2UAS1-p*TEF*-*XlnC*), JMP3097 (*LEU2*ex 3UAS1-p*TEF*-*XlnC*), JMP3098 (*LEU2*ex 4UAS1-p*TEF*-*XlnC*), JMP3099 (*LEU2*ex 8UAS1-p*TEF*-*XlnC*), JMP3100 (*LEU2*ex hp4d-*XlnC*), and JMP3101 (*LEU2*ex hp8d-*XlnC*) plasmids, respectively.

The sequences of the genes encoding YFP and α-amylase are provided in Additional file 3: Data S1. These genes were inserted into pENTR™/D-TOPO^®^ in accordance with the manufacturer’s instructions using the primers listed in Table 1.

The overexpression cassettes, obtained by digesting the plasmids with *Not*I, were used to transform individual strains via the lithium-acetate method [18]. Transformants were selected utilizing YNB Ura, YNB Leu, or YNB medium, depending on their genotype, and their genomic DNA was prepared as described by Querol and colleagues [34]. The primers used to verify expression cassette insertion are given in Table 1.

Restriction enzymes were obtained from OZYME (Saint-Quentin-en-Yvelines, France). PCR was performed using an Eppendorf 2720 thermal cycler; GoTaq DNA polymerases (Promega, Madison, WI, USA) were employed to verify the results and PyroBest DNA polymerases (Takara, Saint-Germain-en-Laye, France) were employed to carry out cloning. PCR and DNA fragment purification were performed as previously described [35]. The amounts of DNA obtained were measured using MySpec (VWR, Fontenay-sous-Bois, France). All the reactions were performed in accordance with the manufacturer’s instructions. The sequencing of the cloned fragments was performed by GATC Biotech (Konstanz, Germany). Clone Manager software was used for the gene sequence analysis (Sci-Ed Software, Morrisville, NC, USA).

### Plasmid pool

Forty ng/µL of each of the recipient plasmids was mixed with pENTR™/D-TOPO^®^ containing the YFP or α-amylase gene. The transfer of the genes of interest was performed using LR Clonase^®^ in accordance with the manufacturer’s instructions (Invitrogen, Saint-Aubin, France). The mixture was used to transform *E. coli* strain DB3.1. The resulting transformants were then pooled, and their DNA was extracted and digested before *Y. lipolytica* was transformed in turn.

### Sds page

Supernatant was obtained from cultures grown for 72 h in YNB, YPD, or YP_2_D_4_ media and was concentrated tenfold in 30 mM Tris (pH 8.0) and 50 mM NaCl using Amicon Ultra-0.5 10 K centrifugal filters (Merck Millipore Ltd, Ireland). Protein production was analyzed via polyacrylamide gel electrophoresis (SDS-PAGE); 4–12% Tris–Glycine gels and an XCell SureLock™ Mini-Cell electrophoresis system (Novex, Life Technologies, Saint-Aubin, France) were used. Prism (MW1; 19–130 kDa) and wide-range (MW2; 14–212 kDa) protein molecular weight markers were used as standards (VWR Chemicals, Fontenay-sous-Bois, France). The gels were stained with 0.2% Coomassie Brilliant Blue R dye (Thermo Fisher Scientific, Villebon-sur-Yvette, France).

### Protein content

Twenty-μL samples were analyzed for protein content using the Coomassie (Bradford) Protein Assay Kit (Thermo Fisher Scientific, Villebon-sur-Yvette, France) in accordance with the manufacturer’s instructions.

### Glucoamylase activity

GA activity was measured as previously described [36], with the following modifications. Samples containing 40 μL of supernatant were incubated for 2–10 min with 1.8 mL of a 0.2% soluble cornstarch solution (30 °C, pH 5). The resulting glucose concentration was determined via high-performance liquid chromatography: an UltiMate^®^ 3000 system (Dionex-Thermo Fisher Scientific, UK) with an Aminex HPX87H column coupled to an RI detector was used. The column was eluted with 0.01 N H_2_SO_4_ at room temperature and a flow rate of 0.6 mL/min. Identification and quantification were achieved via comparison to standards. Enzyme activity was expressed in U mL/L of supernatant, where one unit of GA activity (1 U) was defined as the amount of GA required to release 1 μmol of glucose per minute.

### Xylanase activity

XlnC activity was determined using the EnzChek^®^ Ultra Xylanase Assay Kit (Molecular Probes Invitrogen Ltd., Paisley, UK) in 30 mM Tris (pH 8.0) and 50 mM NaCl at 25 °C in a BioLector^®^ (Biotek, Colmar, France). Prior to the assays, supernatant from cultures grown in YNB medium was diluted 50- and 100-fold, and supernatant from cultures grown in YPD or YP_2_D_4_ was diluted 500- and 1000-fold. As in the case of GA, one unit of XlnC activity (1 U) was defined as the amount of XlnC required to release 1 µmol of xylose per minute.

### Growth analysis

The growth of the *Y. lipolytica* strains was analyzed using a microtiter plate reader, as previously described [37]. RedStar2 fluorescence and YFP fluorescence were analyzed at emission wavelength settings of 558 and 586 nm, respectively; the reception wavelength settings were 505 and 530 nm, respectively.

### Microscopic analysis

Images were acquired using a Zeiss Axio Imager M2 microscope (Zeiss, Le Pecq, France) and Axiovision v. 4.8 software (Zeiss, Le Pecq, France).

### qPCR analysis

RNA extraction was performed using the RNeasy Mini Kit (Qiagen, Courtaboeuf, France) followed by DNA digestion with DNase I (RNase-free; New England BioLabs, Evry, France). cDNA synthesis was performed with the Maxima First Strand cDNA Synthesis Kit with dsDNase (Thermofisher Scientific, Courtaboeuf, France). PCR quantification was performed with CFX Connect™ Real-Time PCR Detection System (Bio-Rad, Marnes-la-Coquette, France) using the SsoAdvanced™ Universal SYBR^®^ Green Supermix Kit (Bio-Rad, Marnes-la-Coquette, France). The number of XlnC mRNA copies was determined using the cycle threshold (Ct) values, which were standardized using results for the expression of the actin gene (YALI0D08272g); the number of XlnC mRNA copies found in the strain containing p*TEF*-*XlnC* was employed as a reference.

## Results and discussion

### RedStar2 expression varies with promoter strength

To examine how protein expression varied with promoter strength, we constructed seven promoters (see diagram in Fig. 1b). Two were conventional promoters: p*TEF* and hp4d. Four new hybrid promoters were generated by combining two, three, four, or eight UAS1 tandem elements taken from hp4d with the *TEF* promoter, yielding 2UAS1-p*TEF*, 3UAS1-p*TEF*, 4UAS1-p*TEF*, and 8UAS1-p*TEF*, respectively (Fig. 1b). We also created a derivative of the hp4d promoter by adding four supplementary UAS1 tandem elements, thus generating hp8d (Fig. 1b).

**Fig. 2:**
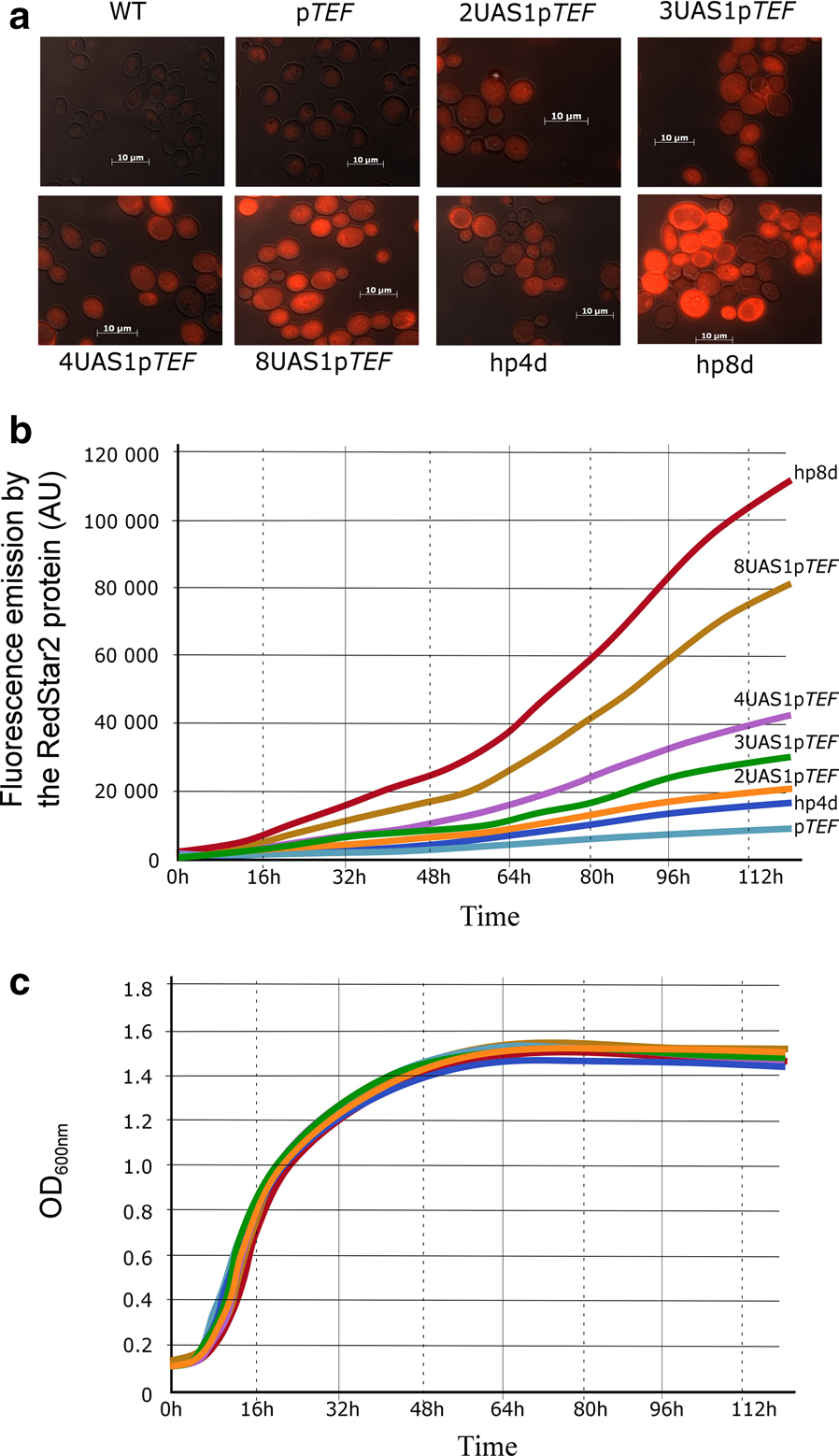
RedStar2 production in the different strains. **a** Microscopic image of the fluorescence patterns of strains overexpressing RedStar2 under one of the seven promoters studied: p*TEF*, 2UAS1-p*TEF*, 3UAS1-p*TEF*, 4UAS1-p*TEF*, 8UAS1-p*TEF*, hp4d, or hp8d. **b** Fluorescence patterns of the different strains overexpressing RedStar2 and cultured in YNB. **c** Growth patterns of the different strains overexpressing RedStar2 and cultured in YNB

Based on previous studies, hp4d and p*TEF* should be the weakest promoters, while hp8d and 8UAS1-p*TEF* should be the strongest. All of these promoters were ligated into a JMP62-*LEU2* plasmid containing the *LEU2* marker and a long-terminal-repeat zeta element that allows random insertion in *Y. lipolytica* (Fig. 1a) [38]. RedStar2 was used as a reporter system to measure promoter strength (Fig. 1c); it was chosen because the protein’s fluorescence is easy to detect and quantify in *Y. lipolytica* [35, 39, 40]. RedStar2 fluorescence was analyzed by microscopy (Fig. 2a) and using a Biotek BioLector^®^ (Fig. 2b, c). Since all the strains showed similar growth patterns (Fig. 2c), their fluorescence levels could be compared. As expected, there was a correlation between putative promoter strength and strain fluorescence (Fig. 2a, b): the stronger the promoter, the greater the fluorescence. Therefore, the strains containing hp4d and p*TEF* had the weakest fluorescence, while the strains containing hp8d and 8UAS1-p*TEF* had the strongest fluorescence. Over time, the fluorescence of strains containing hp8d and 8UAS1-p*TEF* increased 2.3- and 5.3-fold compared to their respective controls, the strains containing hp4d and p*TEF*. Therefore, our results show that increasing the number of UAS1 tandem elements in hybrid promoters resulted in a gradual increase in RedStar2 expression levels (Fig. 2b), confirming the previous findings of Blazeck and colleagues [27, 28]. Thus, our seven promoters varied greatly in strength: there was a 29-fold difference between the weakest (p*TEF*: 4000 AU) and strongest promoter (php8d: 115,000 AU) (Fig. 2b).

### Promoter strength affects xylanase C production but not glucoamylase production

**Fig. 3:**
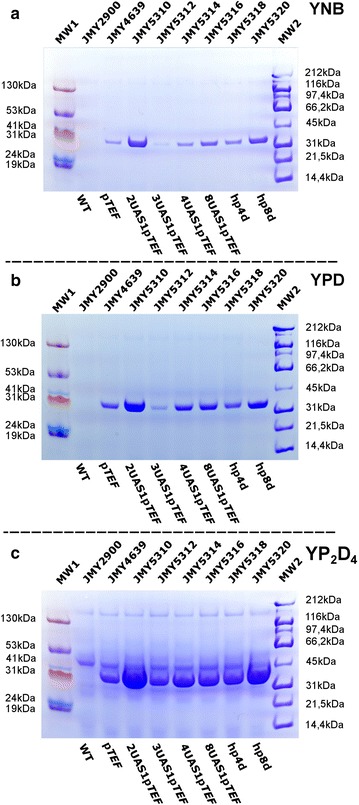
Production of secreted xylanase by the different strains in the different media. SDS-PAGE gel showing xylanase C production by the different strains. **a** YNB medium, **b** YPD medium, and **c** YP_2_D_4_ medium. MW1 and MW2 represent the prism and the wide-range protein molecular weight markers, respectively

We used GA and XlnC to examine how our promoters could be used to produce proteins of industrial interest. GA is used to degrade lignocellulosic materials, the starch in oligosaccharides, or glucose, and it can thus be used by microorganisms to produce biolipids, bioethanol, and other bioindustrial materials [30, 31, 41]. XlnC is a commonly used enzyme in bioprocesses in the paper, textile, and pet-food industries. Therefore, enhancing its production could be of great interest. GA and XlnC activity are also easy to measure (see refs. [42, 43] for GA and “Methods” section for XlnC), making them good candidates for examining the relationship between protein production and promoter strength. To facilitate our analyses (i.e., the visualization of the electrophoresis results and the interpretation of the enzyme assays), the preLip2 secretion sequence was added to the *GA* and *XlnC* genes. This sequence allows proteins to be secreted into the growth medium [6, 14, 30]. Both genes were cloned into different vectors containing the seven different promoters, which were subsequently used to transform *Y. lipolytica*. Cultures were then grown in three media—a defined medium, YNB; a rich medium, YPD; and a very rich medium, YP_2_D_4_—and the levels of secreted GA and XlnC were analyzed (Fig. 3; Additional file 4: Figure S2, Additional file 5: Table S2).

As expected, GA production varied with promoter strength and increased with medium richness (Additional file 4: Figure S2a–d). However, high production levels may or may not translate into high activity levels. To determine if there was a correlation between the two variables, GA activity was estimated by measuring the disappearance of starch and the appearance of glucose. Activity was found to be positively associated with production (Additional file 4: Figure S2e).

**Fig. 4:**
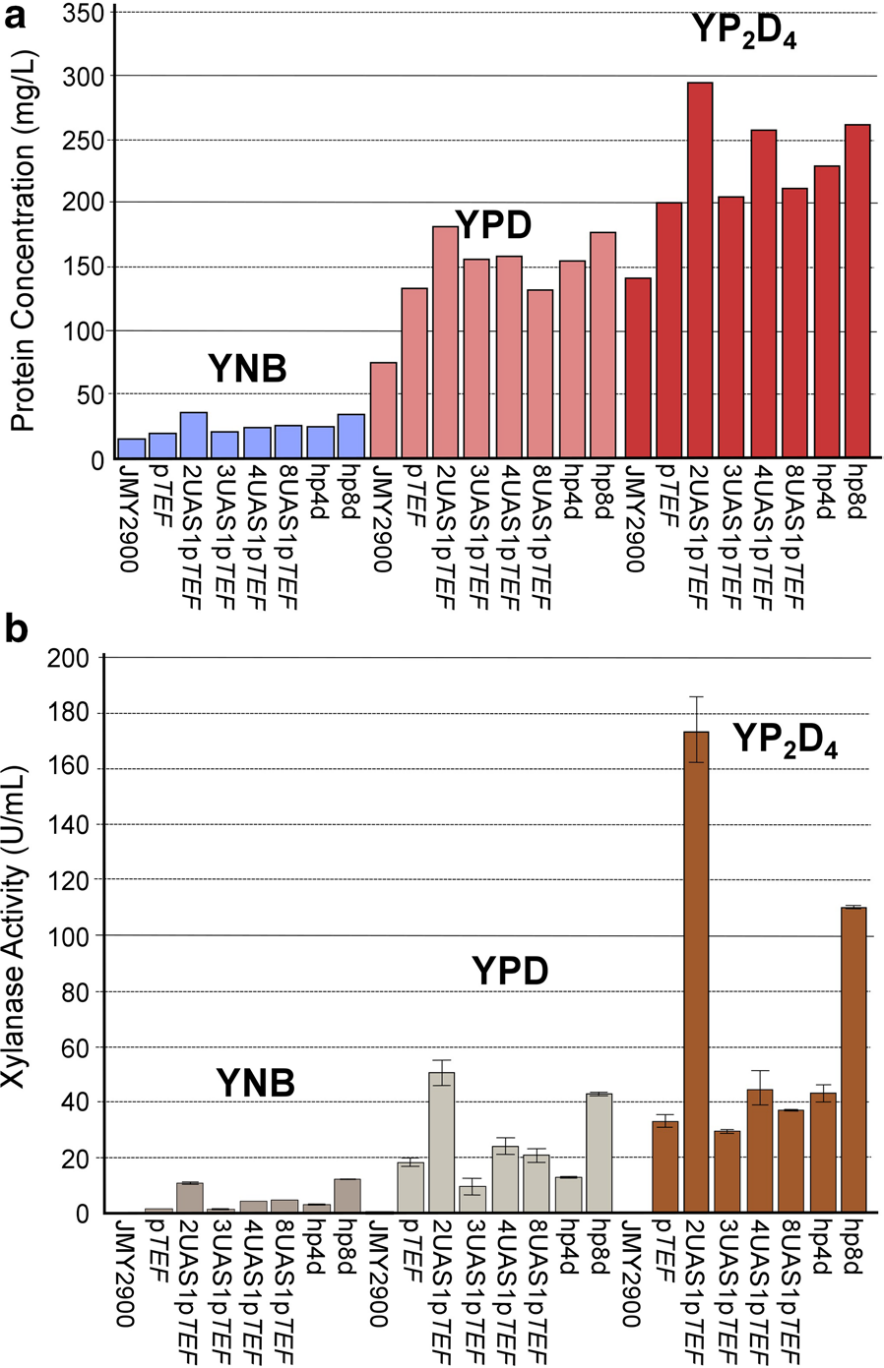
Supernatant protein content and xylanase C activity levels in the different strains in the different media. **a** Total protein content of the supernatant samples containing xylanase C, as assessed by the Bradford assay, for the different strains across the different media (YNB: *blue*; YPD: *pink*; and YP_2_D_4_: *red*). **b** Xylanase C activity in the different strains in the different media (YNB: *dark gray*; YPD: *light gray*; and YP_2_D_4_: *brown*)

**Fig. 5:**
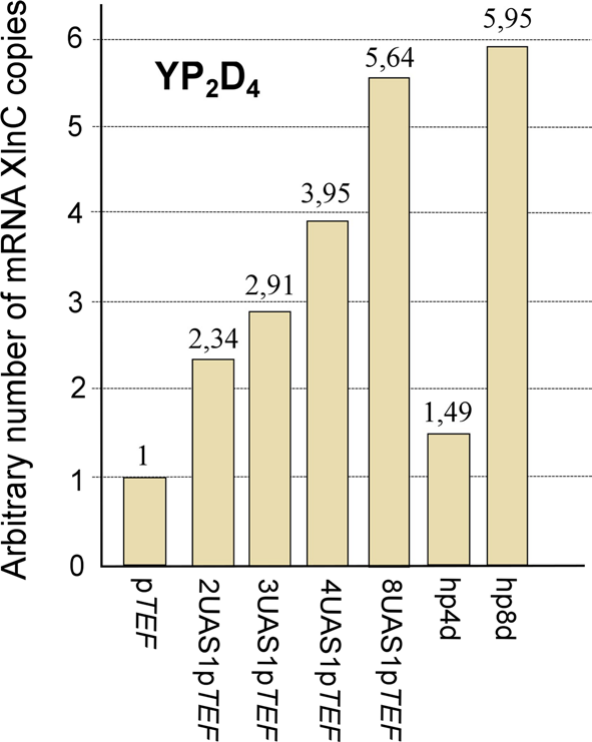
Relationship between *XlnC* transcription and promoter strength. Amount of mRNA produced by strains overexpressing xylanase C grown in YP_2_D_4_ medium. Transcription levels were standardized based on the level observed for the strain containing the p*TEF* promoter

In contrast, XlnC production was not associated with promoter strength. Indeed, across all media, thicker bands were observed for strains containing 2UAS1-p*TEF* and, to a lesser extent, hp8d, whereas band thickness was equivalent for strains containing 3UAS1-p*TEF*, 4UAS1-p*TEF*, 8UAS1-p*TEF*, and hp4d (Figs. 3, 4a; Additional file 5: Table S2). The results were consistent when additional transformants were analyzed. Semi-quantitative PCR confirmed that only one copy of *XlnC* was inserted into the genome of the strain containing 2UAS1-p*TEF* (data not shown). Interestingly, we found that XlnC production was 2–4 times higher in the strain containing 2UAS1-p*TEF* than in the strains containing p*TEF*, 8UAS1-p*TEF*, and hp4d. In our experiment, in YP_2_D_4_, maximum XlnC production was about 153 mg/L. The strain containing 8UAS1-p*TEF* produced slightly more XlnC than the strains containing 3UAS1-p*TEF* and 4UAS1-p*TEF* when the yeasts were cultured in YNB. However, its levels of production were similar or lower when the yeasts were cultured in YPD or YP_2_D_4_. In various microorganisms, several bottlenecks in heterologous protein production have been identified; they include transcription, protein folding and glycosylation, translocation, signal peptide processing, and proteolysis [41–43]. Therefore, several hypotheses could explain why 2UAS1-p*TEF* was the best promoter for XlnC production. To evaluate if this result could be attributed to the 2UAS1-p*TEF* promoter resulting in higher levels of *XlnC* transcription, XlnC mRNA levels were analyzed using qRT-PCR (Fig. 5). However, mRNA levels were positively correlated with promoter strength. This result is consistent with those of a previous study [44], in which researchers observed that the production of an insulin precursor and of amylase was lower under the *TEF1* promoter than under the *TPI* promoter even though their transcription was greater under the *TEF1* promoter. However, it is possible that the use of promoters stronger than 2UAS1-p*TEF* could have resulted in excessive protein production, which could have negatively affected protein folding because of the titration of chaperon proteins and the saturation of secretion machinery, as found previously [43].

As for GA, we examined the correlation between XlnC production and activity (Fig. 4b). As expected, the WT strain, JMY2900, demonstrated no XlnC activity. Surprisingly, activity levels were not always associated with production levels, which could suggest that there was co-secretion of non-active or less-active forms of the enzyme. Although the two variables were correlated when the strains were grown in YNB, the correlation was weak or completely absent when the strains were grown in YP_2_D_4_ or YPD, respectively (Fig. 4a, b). For instance, the strain containing 3UAS1-p*TEF* had a production level similar to that of the strain containing 4UAS1-p*TEF*, but the former’s activity level was much lower. Indeed, its activity level resembled that of the strain containing p*TEF*. Interestingly, activity levels were 1.5–2 times higher than expected for the strains containing 2UAS1-p*TEF* and hp8d (Fig. 4b). Oddly, although these promoters increased protein production two to fourfold, compared to the strain containing p*TEF*, activity increased three to sixfold (Fig. 4a, b; Additional file 5: Table S2, Additional file 6: Table S3). These results underscore that enzyme expression, production, and activity are not always linearly related to promoter strength. Indeed, these relationships may vary and depend on the specific enzyme and growth medium used.

### A gateway vector pool for selecting the best protein producer

Since promoter strength was not necessarily correlated with heterologous protein production, we decided to develop a method for rapidly identifying transformants with optimized production; we used a pool of vectors containing promoters that varied in strength. To simplify the approach, we employed a gateway system that allowed in vitro cloning and the counter-selection of the correct clone using CcdB toxicity. We constructed a derivative of the gateway plasmid JMP1529 described in Leplat et al. [39]: JMP3030 (gateway-*Cla*I-p*TEF*-*BamH*I). Derivatives were constructed using *Cla*I-*Bam*HI-based promoter exchange (Additional file 1: Table S1).

**Fig. 6:**
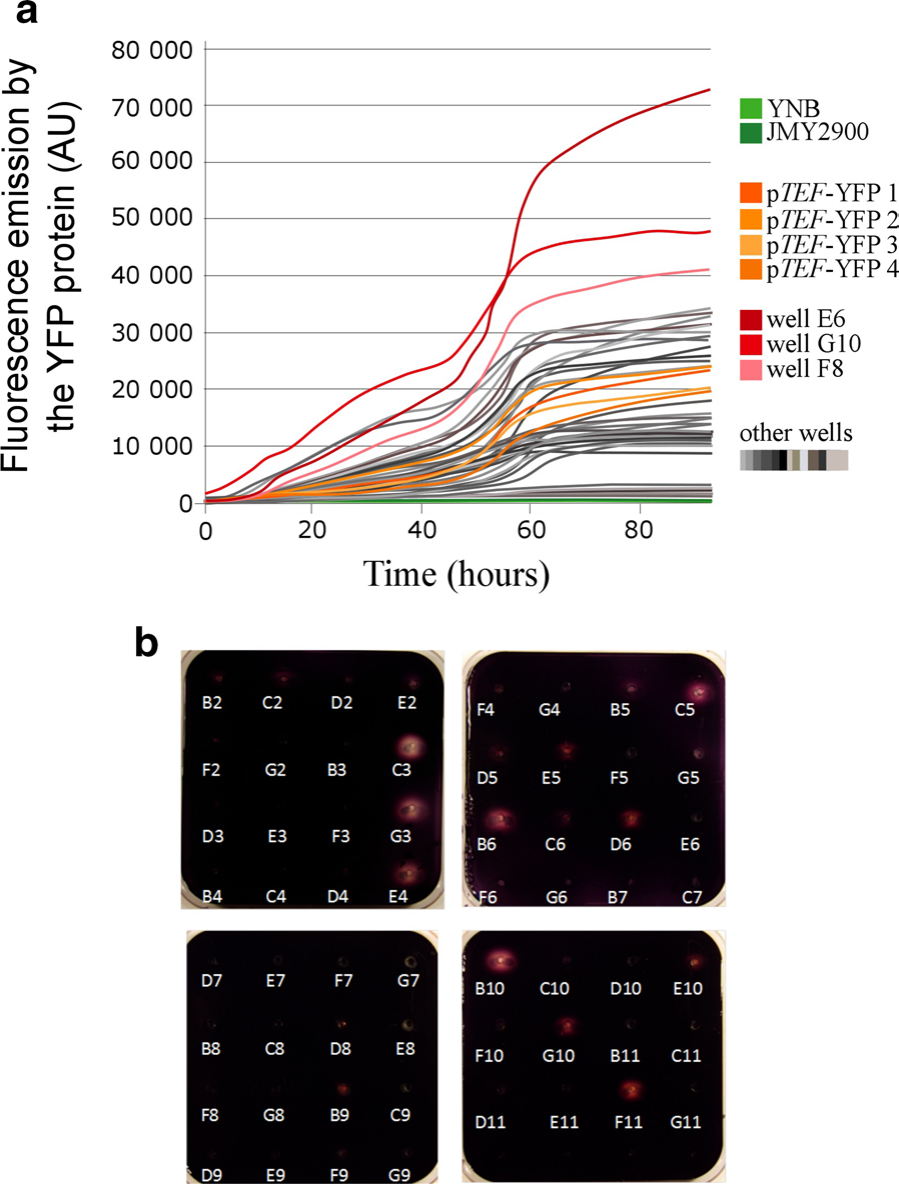
Results of the vector-pool method when used to identify clones with enhanced YFP and α-amylase production. **a** Fluorescence patterns of the strains overexpressing YFP. The figure depicts the negative controls (i.e., the wild-type strain, Y2900, and yeast transformants grown on YNB), the positive controls (the 4 strains overexpressing YFP via p*TEF*), and the 54 strains obtained by the transformation of *Y. lipolytica* via the pool of vectors. The most interesting results are associated with clones E6, G10, and F8. **b** Starch consumption patterns for the strains overexpressing α-amylase. The figure depicts the negative controls (B2: yeast grown on YNB and C2: the wild-type strain, Y2900), the positive controls (D2, E2, F2, and G2: the 4 strains overexpressing α-amylase via the *TEF* promoter), and the 54 strains produced by the transformation of *Y. lipolytica* via the pool of vectors. Starch consumption was measured using iodine crystals

We analyzed the expression of YFP and secreted α-amylase (Fig. 6). Briefly, we first inserted the genes encoding YFP and α-amylase into pENTR™/D-TOPO^®^. We then transferred these genes into a pool of vectors using LR Clonase^®^ (Additional file 7: Figure S3). After transforming *Y. lipolytica*, we analyzed 54 clones for YFP and α-amylase expression (Fig. 6). We found that some clones displayed higher activity levels than others—YFP activity was especially high for the E6, G10, and F8 clones (72,000 U; 48,000 U; and 41,000 U, respectively), and α-amylase activity was especially high for the C3, G3, E4, B6, B10, and F11 clones. Analysis of the promoters involved in the expression of these genes revealed that most of the clones contained a promoter that was stronger than p*TEF* (Table 2). Indeed, with the exception of G10, which contained p*TEF*, the clones contained hp4d, hp8d, 4UAS-p*TEF*, or 8UAS-p*TEF*. However, in some cases, it was difficult to identify the promoter since sequencing was impaired by the multiple UAS1 tandem elements and there was not enough differentiation among fragment sizes to use a PCR-based approach. Therefore, we have proposed two candidate promoters for B6 (α-amylase) and E6 (YFP). Using this method, we have identified several good producers for both enzymes, which shows that it could be very helpful to use a pool of plasmid vectors containing variable-strength promoters to obtain strains that have optimal activity levels.

**Table 2:** Promoters upstream of the α-amylase gene and YFP gene in the different clones

	Promoter
α-Amylase	
C3	4UAS1-p*TEF*
G3	hp4d
E4	hp8d
B6	3UAS1-p*TEF* or 4UAS1-p*TEF*
B10	8UAS1-p*TEF*
F11	8UAS1-p*TEF*
YFP	
E6	4UAS1-p*TEF* or hp8d
F8	8UAS1-p*TEF*
G10	p*TEF*

The clone names are the same as in Fig. 6

## Conclusions

Blazeck and colleagues [27, 28] developed very strong promoters to optimize protein expression in *Y. lipolytica*; however, these promoters were only used to produce intracellular proteins, such as fluorescent proteins, or in β-galactosidase assays. We constructed similar versions of these promoters (p*TEF*, 2UAS1-p*TEF*, 3UAS1-p*TEF*, 4UAS1-p*TEF*, 8UAS1-p*TEF*, hp4d, and hp8d) and analyzed their impact on the production of intracellular proteins, namely RedStar2 and YFP, as well as extracellular proteins, namely glucoamylase, xylanase C, and α-amylase (see summary in Table 3). We found that, most of the time, having the strongest promoter (8UAS-p*TEF*) resulted in the highest levels of protein production and activity (i.e., in the cases of RedStar2, glucoamylase, YFP, and α-amylase). However, the best promoters for xylanase C were 2UAS1-p*TEF* and hp8d. Our results show that stronger promoters do not always optimize protein production and activity. It could be that either transcriptional or post-translational regulation, such as RNA processing and stability, translation efficiency, or protein stability and modification [45, 46], places limits on this relationship. As a result, multiple promoters should always be tested. To limit clone number and keep the process simple, we developed a straightforward strategy for accomplishing this aim: exploiting a pool of vectors containing promoters of different strengths. Cloning was facilitated by using the gateway system and LR Clonase^®^. Indeed, in a single step, it was possible to obtain a collection of vectors containing variable-strength promoters upstream from the gene of interest. Once the study organism has been transformed, screening tests can be used to select the best strains. This approach could be very helpful to those seeking to improve protein production, whether in a research or an industrial setting. The pool should contain a decent number of promoters and include inducible promoters, which could be particularly important when dealing with toxic proteins.

**Table 3:** Relative results for the experiments examining RedStar2, glucoamylase, and xylanase C expression under the seven different promoters studied

	Redstar2	GA		XlnC		
	Activity	Production	Activity	RNA	Production	Activity
p*TEF*	+	+	+	+	+	+
2UAS-p*TEF*	++	++	++	++	+++++	+++++
3UAS-p*TEF*	+++	++	++	+++	±	+
4UAS-p*TEF*	++++	+++	+++	++++	+	+
8UAS-p*TEF*	+++++	++++	++++	+++++	+++	+
hp4d	++	+	++	+	++	+
hp8d	+++++	+++	++++	+++++	++++	+++

The number of crosses indicate very low (±), low (+), medium (++), high (+++), very high (++++) and extremely high (+++++) levels

## Additional files

**Additional file 1: Table S1.** Details on the strains and plasmids used in this study.

**Additional file 2: Figure S1.** Schematic representation of plasmid and strain construction.

**Additional file 3: Data S1.** Sequences of the genes used in this study.

**Additional file 4: Figure S2.** Production and activity of secreted glucoamylase for the different strains in the different media.

**Additional file 5: Table S2.** Glucoamylase and xylanase C concentrations.

**Additional file 6: Table S3.** Xylanase activity levels.

**Additional file 7: Figure S3.** Schematic representation of the construction of the vector pool.

## Abbreviations

GA: glucoamylase
XlnC: xylanase C
YFP: yellow fluorescent protein
PCR: polymerase chain reaction
