# Supplementary material for: Using a vector pool containing variable-strength promoters to optimize protein production in Yarrowia lipolytica
Source: Microb Cell Fact. 2017 Feb 17;16:31. doi: 10.1186/s12934-017-0647-3 (PMC5316184; doi:10.1186/s12934-017-0647-3)
Supplement: Supplementary file 1 — Additional file 1: Table S1. Details on the strains and plasmids used in this study. [file 12934_2017_647_MOESM1_ESM.docx]

**Additional Table S1.** Strains and plasmids

| Strains | Genotype or other relevant characteristics | Source or reference |
| --- | --- | --- |
| ***E. coli*** |  |  |
| DH5α | *Φ80dlacZ*Δ*m15, recA1, endA1, gyrA96, thi-1, hsdR17 (r_k_−, m_k_+), supE44, relA1, deoR,* Δ*(lacZYA-argF)U169* | Thermo Fisher Scientific, Asnières, France |
| DB3.1 | *gyrA462*, *endA1*, ∆(*sr1*-*recA*), *mcrB*, *mrr*, *hsdS20*, *glnV44* (=*supE44*), *ara14*, *galK2*, *lacY1*, *proA2*, *rpsL20*, *xyl5*, *leuB6*, *mtl1* | Thermo Fisher Scientific, Asnières, France |
|  |  |  |
| ***Y. lipolytica*** |  |  |
| W29 | *MATA, wild-type* | (1) |
| Po1d | *MATA ura*3-302 *leu*2-270 *xpr*2-322 | (1) |
| JMY330 | Po1d Ura+ Leu- | (2) |
| JMY2101 | Po1d Ura- Leu+ | This study |
| JMY2900 | Po1d Ura+ Leu+ | (3) |
| JMY5083 | JMY330 *LEU2ex*-p*TEF*-Glucoamylase | (4) |
| JMY5310 | JMY330 *LEU2ex*-2UAS1-p*TEF*-*XlnC* | This study |
| JMY5312 | JMY330 *LEU2ex*-3UAS1-p*TEF*-*XlnC* | This study |
| JMY5314 | JMY330 *LEU2ex*-4UAS1-p*TEF*-*XlnC* | This study |
| JMY5316 | JMY330 *LEU2ex*-8UAS1-p*TEF*-*XlnC* | This study |
| JMY5318 | JMY330 *LEU2ex*-hp4d-*XlnC* | This study |
| JMY5320 | JMY330 *LEU2ex*-hp8d-*XlnC* | This study |
| JMY5771 | JMY330 *LEU2ex*-2UAS1-p*TEF*-*GA* | This study |
| JMY5774 | JMY330 *LEU2ex*-3UAS1-p*TEF*-*GA* | This study |
| JMY5777 | JMY330 *LEU2ex*-4UAS1-p*TEF*-*GA* | This study |
| JMY5780 | JMY330 *LEU2ex*-8UAS1-p*TEF*-*GA* | This study |
| JMY5783 | JMY330 *LEU2ex*-hp4d-*GA* | This study |
| JMY5785 | JMY330 *LEU2ex*-hp8d-*GA* | This study |
| JMY6313 | JMY784 *URA3ex*-p*TEF*-*XlnC* | This study |
| JMY6386 | JMY330 *LEU2-*p*TEF*-Redstar2 | This study |
| JMY6387 | JMY330 *LEU2-*2UAS1-p*TEF*-Redstar2 | This study |
| JMY6388 | JMY330 *LEU2-*3UAS1-p*TEF*-Redstar2 | This study |
| JMY6388 | JMY330 *LEU2*-4UAS1-p*TEF*-Redstar2 | This study |
| JMY6389 | JMY330 *LEU2* 8UAS1-p*TEF*-Redstar2 | This study |
| JMY6390 | JMY330 *LEU2-*hp4d-Redstar2 | This study |
| JMY6391 | JMY330 *LEU2*-hp8d-Redstar2 | This study |
|  |  |  |
| **Plasmides** |  |  |
| pCR4Blunt-TOPO® | Cloning vector | Thermo Fisher Scientific, Asnières, France |
| pENTR™/D-TOPO® | Cloning vector | Thermo Fisher Scientific, Asnières, France |
| JMP1047 | JMP62 *URA3ex*-p*TEF* | (5) |
| JMP1394 | JMP62 *LEU2ex* p*TEF*-RedStar2 | (6) |
| JMP1521 | JMP62 p*TEF* *URA3ex* AmpR and ORI from PBR322 | (7) |
| JMP1524 | JMP62 *URA3ex*-hp4d | Verbeke J. unpublished |
| JMP1529 | JMP1521-cassette *ccdB* (Gateway) | (7) |
| JMP2416 | pCR4Blunt-TOPO - *ClaI*-2UAS1-*BstBI* | This study |
| JMP2418 | pCR4Blunt-TOPO - *ClaI*-3UAS1-*BstBI* | This study |
| JMP2027 | pCR4Blunt-TOPO - *ClaI*-4UAS1-*BstBI* | This study |
| JMP2397 | JMP62 *LEU2ex*-4UAS1-p*TEF*-RedStar2 | This study |
| JMP2471 | JMP62 *LEU2ex*-hp4d -RedStar2 | This study |
| JMP2473 | JMP62 *LEU2ex*-hp8d -RedStar2 | This study |
| JMP2482 | JMP62 *LEU2ex*-2UAS1-p*TEF*-RedStar2 | This study |
| JMP2484 | JMP62 *LEU2ex*-3UAS1-p*TEF*-RedStar2 | This study |
| JMP2563 | JMP62 *LEU2ex*-p*TEF* | Crutz-Le Coq A.M., Fouchard F., unpublished |
| JMP2603 | JMP62 *URA3ex*-p*TEF*-*XlnC* | This study |
| JMP2607 | JMP62 *LEU2ex*-8UAS1-p*TEF*-RedStar2 | This study |
| JMP2928 | JMP62 *URA3ex*-p*TEF*-Glucoamylase | (4) |
| JMP3030 | p1529 + *Cla*I/*Bam*HI p*TEF* | This study |
| JMP3048 | p3030 8UAS1-p*TEF* | This study |
| JMP3050 | p3030 hp8d clone 7 | This study |
| JMP3052 | p3030 4UAS1-p*TEF* | This study |
| JMP3096 | JMP62 *LEU2ex*-2UAS1-p*TEF*-*XlnC* | This study |
| JMP3097 | JMP62 *LEU2ex*-3UAS1-p*TEF*-*XlnC* | This study |
| JMP3098 | JMP62 *LEU2ex*-4UAS1-p*TEF*-*XlnC* | This study |
| JMP3099 | JMP62 *LEU2ex*-8UAS1-p*TEF*-*XlnC* | This study |
| JMP3100 | JMP62 *LEU2ex*-hp4d-*XlnC* | This study |
| JMP3101 | JMP62 *LEU2ex*-hp8d-*XlnC* | This study |
| JMP3258 | p3030 2UAS1-p*TEF* | This study |
| JMP3273 | p3030 3UAS1-p*TEF* | This study |
| JMP3274 | p3030 3UAS1-p*TEF* | This study |
| JMP3275 | p3030 hp4d | This study |
| JMP3276 | p3030 hp4d | This study |
| JMP3781 | JMP62 *LEU2ex*-2UAS1-p*TEF*-Glucoamylase | This study |
| JMP3782 | JMP62 *LEU2ex*-3UAS1-p*TEF*-Glucoamylase | This study |
| JMP3783 | JMP62 *LEU2ex*-4UAS1-p*TEF*-Glucoamylase | This study |
| JMP3784 | JMP62 *LEU2ex*-8UAS1-p*TEF*-Glucoamylase | This study |
| JMP3785 | JMP62 *LEU2ex*-hp4d-Glucoamylase | This study |
| JMP3786 | JMP62 *LEU2ex*-hp8d-Glucoamylase | This study |

References

1. Barth G, Gaillardin C. *Yarrowia lipolytica*. In : Wolf, K. editors, Non conventional yeasts in biotechnology. Vol.1, Springer, Berlin, Germany; 1996. p. 313–388.
2. Haddouche R, Delessert S, Sabirova J, Neuvéglise C, Poirier Y, Nicaud JM. Roles of multiple acyl-CoA oxidases in the routing of carbon flow towards β-oxidation and polyhydroxyalkanoate biosynthesis in *Yarrowia lipolytica*. FEMS Yeast Res. 2010;10: 917-927.
3. Dulermo R, Gamboa-Meléndez H, Dulermo T, Thevenieau F, Nicaud JM. The fatty acid transport protein Fat1p is involved in the export of fatty acids from lipid bodies in *Yarrowia lipolytica*. FEMS Yeast Res. 2014;14: 883-896.
4. Ledesma-Amaro R, Dulermo T, Nicaud JM. Engineering *Yarrowia lipolytica* to produce biodiesel from raw starch. Biotechnol Biofuels. 2015;8: 148.
5. Lazar Z, Rossignol T, Verbeke J, Crutz-Le Coq AM, Nicaud JM, Robak M. Optimized invertase expression and secretion cassette for improving *Yarrowia lipolytica* growth on sucrose for industrial applications. J Ind Microbiol Biotechnol. 2013;40: 1273-1283.
6. Dulermo R, Gamboa-Meléndez H, Ledesma-Amaro R, Thévenieau F, Nicaud JM. Unraveling fatty acid transport and activation mechanisms in *Yarrowia lipolytica*. Biochim Biophys Acta. 2015;1851: 1202-1217.
7. Leplat C, Nicaud JM, Rossignol T. High-throughput transformation method for *Yarrowia lipolytica* mutant library screening. FEMS Yeast Res. 2015;15: pii: fov052.
