# Supplementary material for: Using a vector pool containing variable-strength promoters to optimize protein production in Yarrowia lipolytica
Source: Microb Cell Fact. 2017 Feb 17;16:31. doi: 10.1186/s12934-017-0647-3 (PMC5316184; doi:10.1186/s12934-017-0647-3)
Supplement: Supplementary file 2 — Additional file 2: Figure S1. Schematic representation of plasmid and strain construction. [file 12934_2017_647_MOESM2_ESM.pptx]

## Slide 1
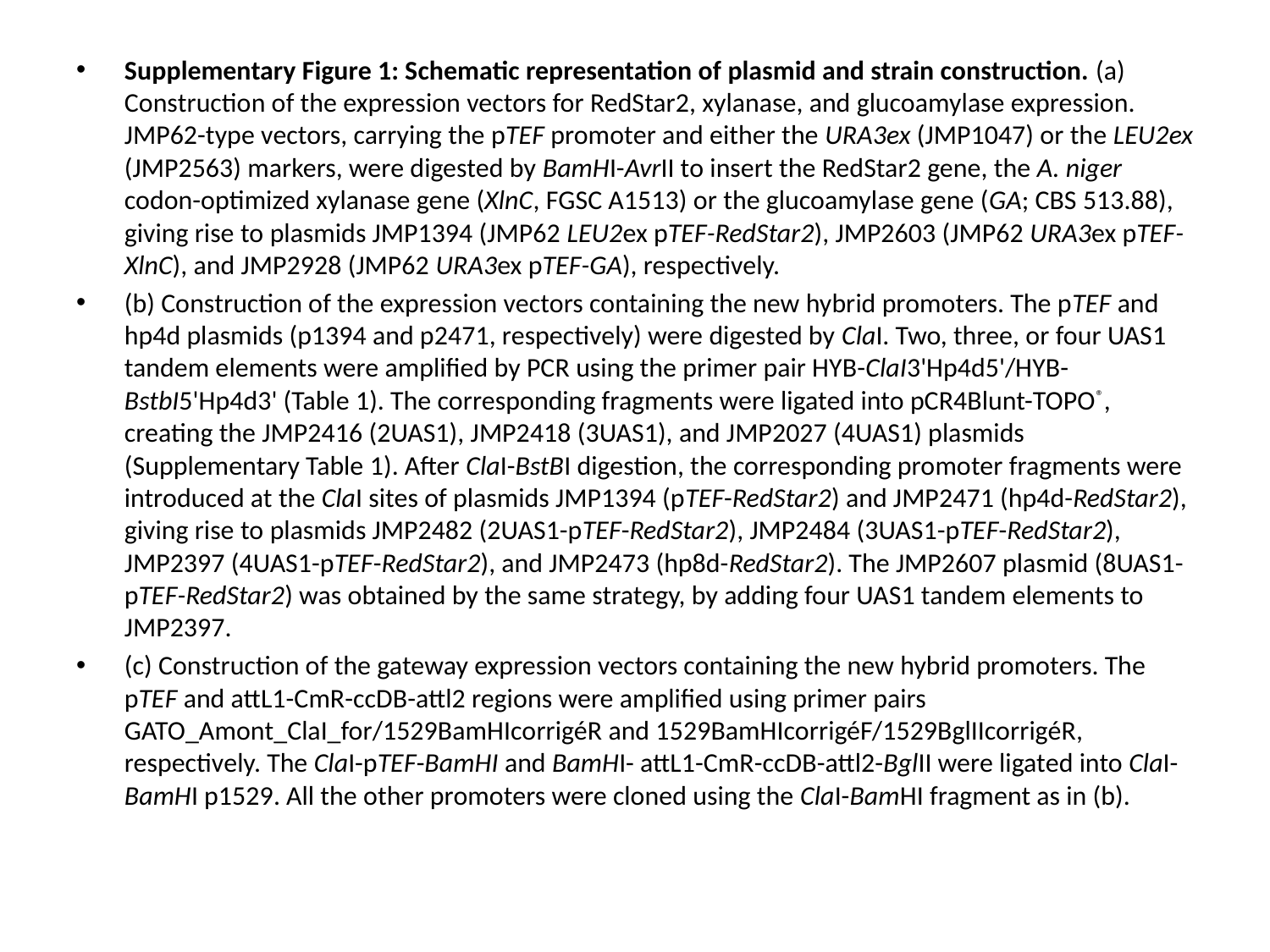

#
Supplementary Figure 1: Schematic representation of plasmid and strain construction. (a) Construction of the expression vectors for RedStar2, xylanase, and glucoamylase expression. JMP62-type vectors, carrying the pTEF promoter and either the URA3ex (JMP1047) or the LEU2ex (JMP2563) markers, were digested by BamHI-AvrII to insert the RedStar2 gene, the A. niger codon-optimized xylanase gene (XlnC, FGSC A1513) or the glucoamylase gene (GA; CBS 513.88), giving rise to plasmids JMP1394 (JMP62 LEU2ex pTEF-RedStar2), JMP2603 (JMP62 URA3ex pTEF-XlnC), and JMP2928 (JMP62 URA3ex pTEF-GA), respectively.
(b) Construction of the expression vectors containing the new hybrid promoters. The pTEF and hp4d plasmids (p1394 and p2471, respectively) were digested by ClaI. Two, three, or four UAS1 tandem elements were amplified by PCR using the primer pair HYB-ClaI3'Hp4d5'/HYB-BstbI5'Hp4d3' (Table 1). The corresponding fragments were ligated into pCR4Blunt-TOPO®, creating the JMP2416 (2UAS1), JMP2418 (3UAS1), and JMP2027 (4UAS1) plasmids (Supplementary Table 1). After ClaI-BstBI digestion, the corresponding promoter fragments were introduced at the ClaI sites of plasmids JMP1394 (pTEF-RedStar2) and JMP2471 (hp4d-RedStar2), giving rise to plasmids JMP2482 (2UAS1-pTEF-RedStar2), JMP2484 (3UAS1-pTEF-RedStar2), JMP2397 (4UAS1-pTEF-RedStar2), and JMP2473 (hp8d-RedStar2). The JMP2607 plasmid (8UAS1-pTEF-RedStar2) was obtained by the same strategy, by adding four UAS1 tandem elements to JMP2397.
(c) Construction of the gateway expression vectors containing the new hybrid promoters. The pTEF and attL1-CmR-ccDB-attl2 regions were amplified using primer pairs GATO_Amont_ClaI_for/1529BamHIcorrigéR and 1529BamHIcorrigéF/1529BglIIcorrigéR, respectively. The ClaI-pTEF-BamHI and BamHI- attL1-CmR-ccDB-attl2-BglII were ligated into ClaI-BamHI p1529. All the other promoters were cloned using the ClaI-BamHI fragment as in (b).

## Slide 2
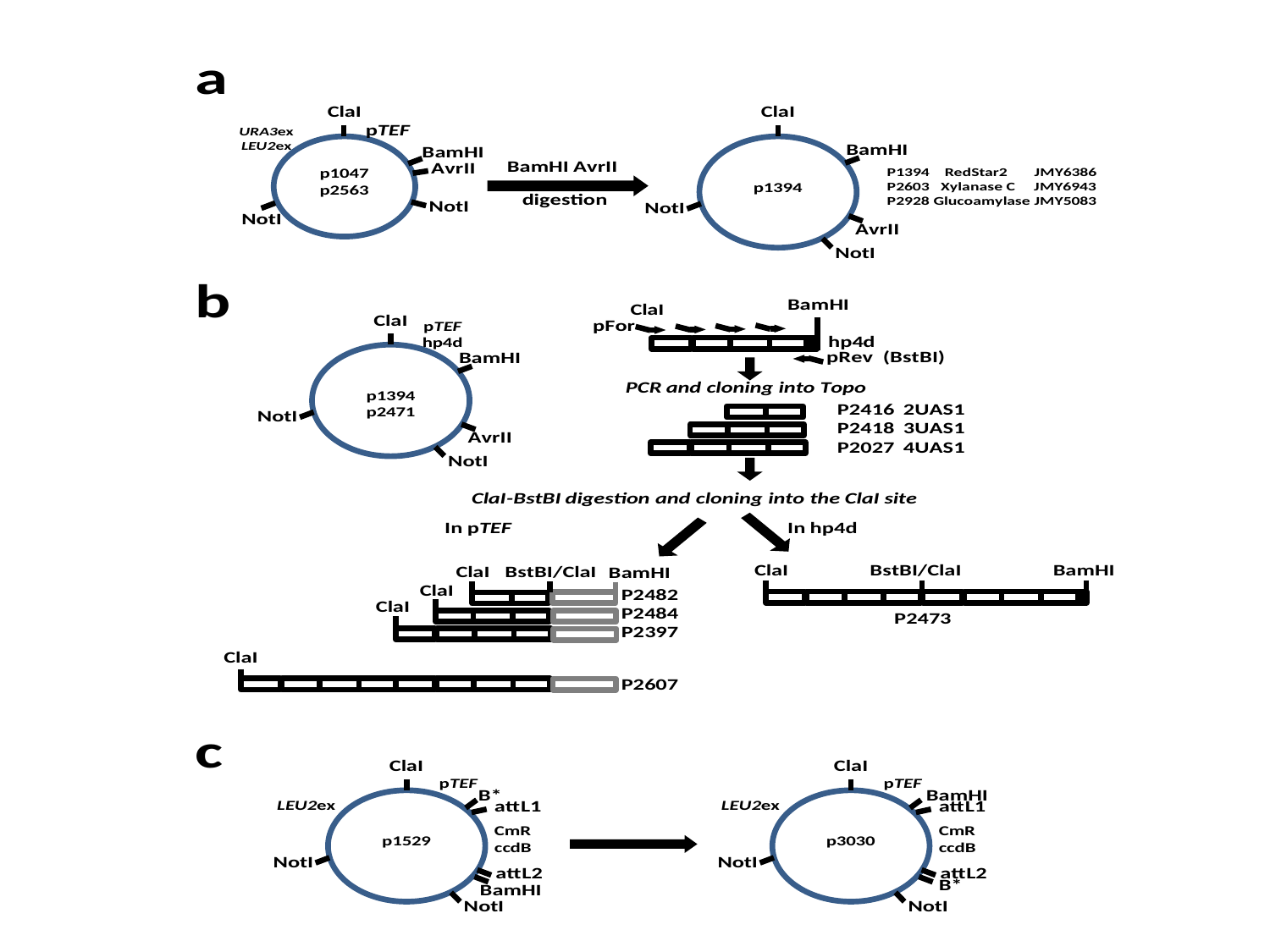

#
