# Supplementary material for: Using a vector pool containing variable-strength promoters to optimize protein production in Yarrowia lipolytica
Source: Microb Cell Fact. 2017 Feb 17;16:31. doi: 10.1186/s12934-017-0647-3 (PMC5316184; doi:10.1186/s12934-017-0647-3)

**Supplementary Figure 2:** **Production and activity of secreted glucoamylase for the different strains in the different media.** Production of GA by strains grown in (a) YNB medium, (b) YPD medium, and (c) YP_2_D_4_ medium. MW1 and MW2 represent the prism and the wide-range protein molecular weight markers, respectively. (d) Total protein content of the supernatant samples containing glucoamylase, as assessed by the Bradford assay, for the different strains in the different media (YNB: blue; YPD: pink; and YP_2_D_4_: red). (e) Glucoamylase activity in the different strains in the different media (YNB: dark gray; YPD: light gray; and YP_2_D_4_: brown).


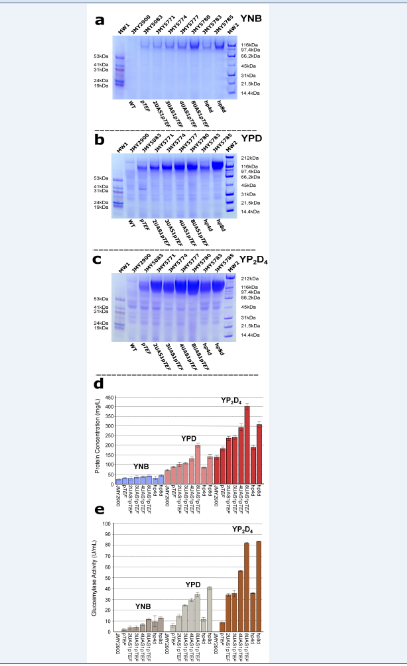

Supplement: Supplementary file 4 — Additional file 4: Figure S2. Production and activity of secreted glucoamylase for the different strains in the different media. [file 12934_2017_647_MOESM4_ESM.docx]
