# Supplementary material for: Using a vector pool containing variable-strength promoters to optimize protein production in Yarrowia lipolytica
Source: Microb Cell Fact. 2017 Feb 17;16:31. doi: 10.1186/s12934-017-0647-3 (PMC5316184; doi:10.1186/s12934-017-0647-3)

**Supplementary Figure 3: Schematic representation of the construction of the vector pool.** The gene of interest was cloned into the donor vector pENTR^™^/D-TOPO^®^, which was then transferred into the gateway pool of recipient vectors (i.e., consisting of JMP62-derived plasmids containing different vectors) using LR Clonase^®^. The LR reaction was used to transform *E. coli*. DNA was then extracted from the *E. coli* pool and digested by *Not*I. The resulting pool of expression cassettes was used to transform *Y. lipolytica*.


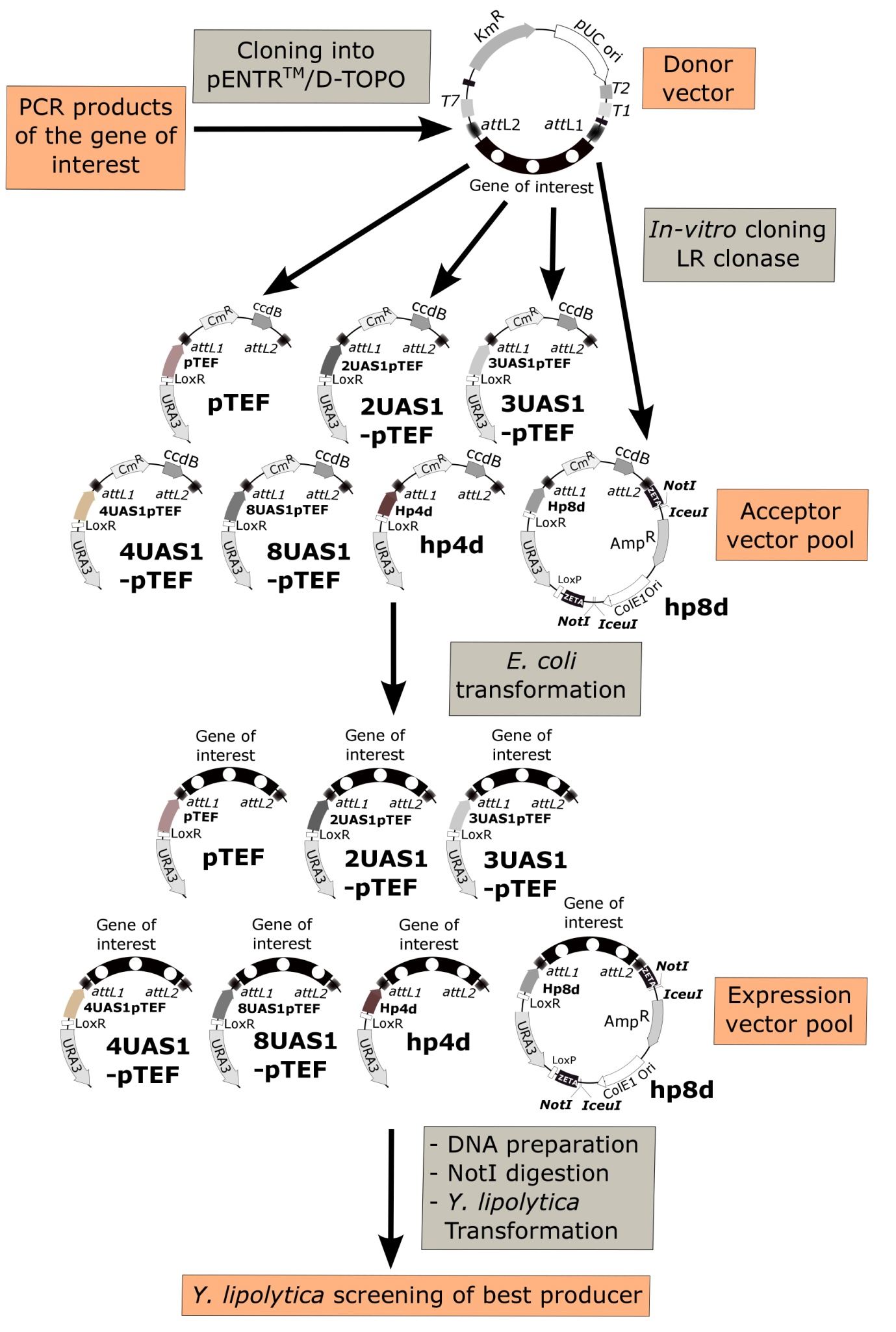

Supplement: Supplementary file 7 — Additional file 7: Figure S3. Schematic representation of the construction of the vector pool. [file 12934_2017_647_MOESM7_ESM.docx]
